# Radiation-induced malignancies after intensity-modulated versus conventional mediastinal radiotherapy in a small animal model

**DOI:** 10.1038/s41598-019-51735-3

**Published:** 2019-10-29

**Authors:** Kaga Gomarteli, Jens Fleckenstein, Stefanie Kirschner, Vladimir Bobu, Marc A. Brockmann, Thomas Henzler, Mathias Meyer, Philipp Riffel, Stefan O. Schönberg, Marlon R. Veldwijk, Bettina Kränzlin, Christian Hoerner, Gerhard Glatting, Frederik Wenz, Carsten Herskind, Frank A. Giordano

**Affiliations:** 1 Department of Radiation Oncology, Universitätsmedizin Mannheim, Medical Faculty Mannheim, University of Heidelberg, Mannheim, Germany; 2 Department of Neuroradiology, University Medical Center Mainz, Johannes Gutenberg-University, Mainz, Germany; 3 Department of Clinical Radiology and Nuclear Medicine, Universitätsmedizin Mannheim, Medical Faculty Mannheim, University of Heidelberg, Mannheim, Germany; 4 Medical Research Center, Universitätsmedizin Mannheim, Medical Faculty Mannheim, University of Heidelberg, Mannheim, Germany; 5 Institute of Pathology, Universitätsmedizin Mannheim, Medical Faculty Mannheim, University of Heidelberg, Mannheim, Germany; 6 Medical Radiation Physics, Department of Nuclear Medicine, Ulm University, Ulm, Germany

**Keywords:** Translational research, Cancer prevention, Cancer models

## Abstract

A long-standing hypothesis in radiotherapy is that intensity-modulated radiotherapy (IMRT) increases the risk of second cancer due to low-dose exposure of large volumes of normal tissue. Therefore, young patients are still treated with conventional techniques rather than with modern IMRT. We challenged this hypothesis in first-of-its-kind experiments using an animal model. Cancer-prone *Tp53*^+/C273X^ knockout rats received mediastinal irradiation with 3 × 5 or 3 × 8 Gy using volumetric-modulated arc therapy (VMAT, an advanced IMRT) or conventional anterior-posterior/posterior-anterior (AP/PA) beams using non-irradiated rats as controls (*n* = 15/group, *n*_*total*_ = 90). Tumors were assigned to volumes receiving 90–107%, 50–90%, 5–50%, and <5% of the target dose and characterized by histology and loss-of-heterozygosity (LOH). Irradiated rats predominantly developed lymphomas and sarcomas in areas receiving 50–107% (*n* = 26) rather than 5–50% (*n* = 7) of the target dose. Latency was significantly shortened only after 3 × 8 Gy vs. controls (*p* < 0.0001). The frequency (14/28 vs. 19/29; *p* = 0.29) and latency (218 vs. 189 days; *p* = 0.17) of radiation-associated tumors were similar after VMAT vs. AP/PA. LOH was strongly associated with sarcoma but not with treatment. The results do not support the hypothesis that IMRT increases the risk of second cancer. Thus the current practice of withholding dose-sparing IMRT from young patients may need to be re-evaluated.

## Introduction

Long-term cancer survivors have an increased risk of developing second cancer (SC) after treatment for their primary cancer^1,2^. An epidemiological study on adult cancer patients in the United States estimated a total of 3,266 excess SCs attributed to radiotherapy in 485,481 cancer survivors treated with radiotherapy for their first disease^3^. However, children and adolescent patients (e. g. young patients with Hodgkin’s Lymphoma) have a considerably higher risk for SC after cancer therapy than adults, owing to stimulation of proliferation of stem cells and early progenitor cells during growth and puberty^4–6^.

A widely accepted dogma in radiotherapy is that intensity-modulated radiotherapy (IMRT) may double the risk of SC compared to three-dimensional conformal radiotherapy (3D-CRT)^7^. As a consequence, young patients are less likely to be treated with IMRT and are, therefore, exposed to a higher risk than older patients for developing other adverse normal-tissue reactions. Because classic models predict a linear increase in risk at low doses reaching a maximum or a plateau at 4–8 Gray (Gy)^8^, the estimated risk would be expected to increase with the volume exposed to low-dose irradiation. However, secondary carcinomas and sarcomas are predominantly found within and near the high-dose volume^9,10^. Furthermore, a linear increase in risk with dose up to integral radiation doses >10 Gy was reported for induction of bone and soft tissue sarcomas in patients treated in childhood with radiotherapy only^4,11^. Therefore, the assumptions underlying the predicted doubling of the SC risk for IMRT may not hold true in clinical practice.

The purpose of the present study was to compare the rates of radiation-induced tumors in an experimental model after irradiation with a modern, highly conformal rotational volumetric modulated arc therapy (VMAT, an advanced version of IMRT) or a conventional 3D-CRT technique with two opposing anterior-posterior/posterior-anterior (AP/PA) beams. Cancer-prone rats, heterozygous for the *Tumor protein 53* (*Tp53*), were irradiated with three fractions of high-energy X-rays to the mediastinum, and the development of tumors in the high- and low-dose regions was compared.

## Methods

### Animal model

The present study was carried out in accordance with the European Union (EU) Directive 2010/63/EU for animal welfare and experimental conduct after approval by the regional animal care committee (‘Regierungspräsidium Karlsruhe’, Germany; approval number: 35-9185 81/G184/14). Heterozygous *Tp53*^+/C273X^ knockout rats (Crl:WI(UL)-Tp53^m1/Hubr^)^12^ established by target-selected mutagenesis^13^, were kindly obtained from Dr. Edwin Cuppen (Hubrecht Institute for Developmental Biology and Stem Cell Research, Utrecht, The Netherlands). A nonsense mutation in codon 273 (C273) in exon 6 of the rat *Tp53* gene (homologous to codon 275 in exon 8 of the human *TP53* gene) produces no detectable truncated protein 53 (p53) in rats homozygous for this mutation, probably as a result of nonsense-mediated mRNA decay^12^. Heterozygous *Tp53*^+/C273X^ rats were chosen as a model because of the longer latency time for tumor development compared to homozygous animals. The model is similar but not identical to the classic Li-Fraumeni syndrome in humans, which is usually characterized by heterozygosity for an inactivating amino acid substitution in *TP53*^14^.

Animal genotyping was performed on DNA extracts from ear biopsies. *Tp53* status and loss of heterozygosity (LOH) in genomic DNA extracts were tested by polymerase chain reaction (PCR) and Sanger sequencing (Eurofins GATC Biotech, Konstanz, Germany). Primer design and further experimental details are given in Supplementary Information (Supplementary Methods and Supplementary Fig. S1).

### Animal care and treatment groups

Animals were housed under controlled conditions (12-hour light/dark cycle, 22 ± 2 °C, 45% relative humidity, food and water *ad libitum*). Quarterly pathogen screening by a veterinary laboratory revealed no new pathogens in animals during the entire husbandry period.

Experimental *Tp53*^+/C273X^ rats were derived from 2 male and 3 female *Tp53*^+/C273X^ ancestors for filial generation 1 (F1) rat litters 1 to 3 (L1-L3) and from six pairs of homozygous (*Tp53*^C273X/C273X^) male and wild type female F1 non-siblings generating F2 litters (L4-L14). The low number of ancestors helped limit heterogeneity of the genetic background, and using F1 homozygous males allowed a reduction of the total number of animals required compared with using heterozygous parents. Siblings from each litter were randomly allocated to six treatment groups, anesthesia only (AN), positioning cone-beam computed tomography (CBCT), VMAT 3 × 5 Gy, AP/PA 3 × 5 Gy, VMAT 3 × 8 Gy, and AP/PA 3 × 8 Gy (*n* = 15 per group), to balance ancestral background between the groups (Supplementary Table S1). The distribution of sex, weight and age (gender-mixed) were also balanced for rats recruited to the VMAT or AP/PA groups, however, the median age at treatment was 37.5 days higher for the two groups irradiated with 3 × 8 Gy (106 days) compared with the other groups (68.5 days) (Supplementary Table S2 and Supplementary Fig. S2). The potential influence of this difference on the results was considered in the analysis of the data.

Before any experiment, all animals received anesthesia by subcutaneous injection of 0.15 mg medetomidine, 2.0 mg midazolam, and 0.005 mg fentanyl per kg body mass.

### Treatment planning and irradiation

**Figure 1:**
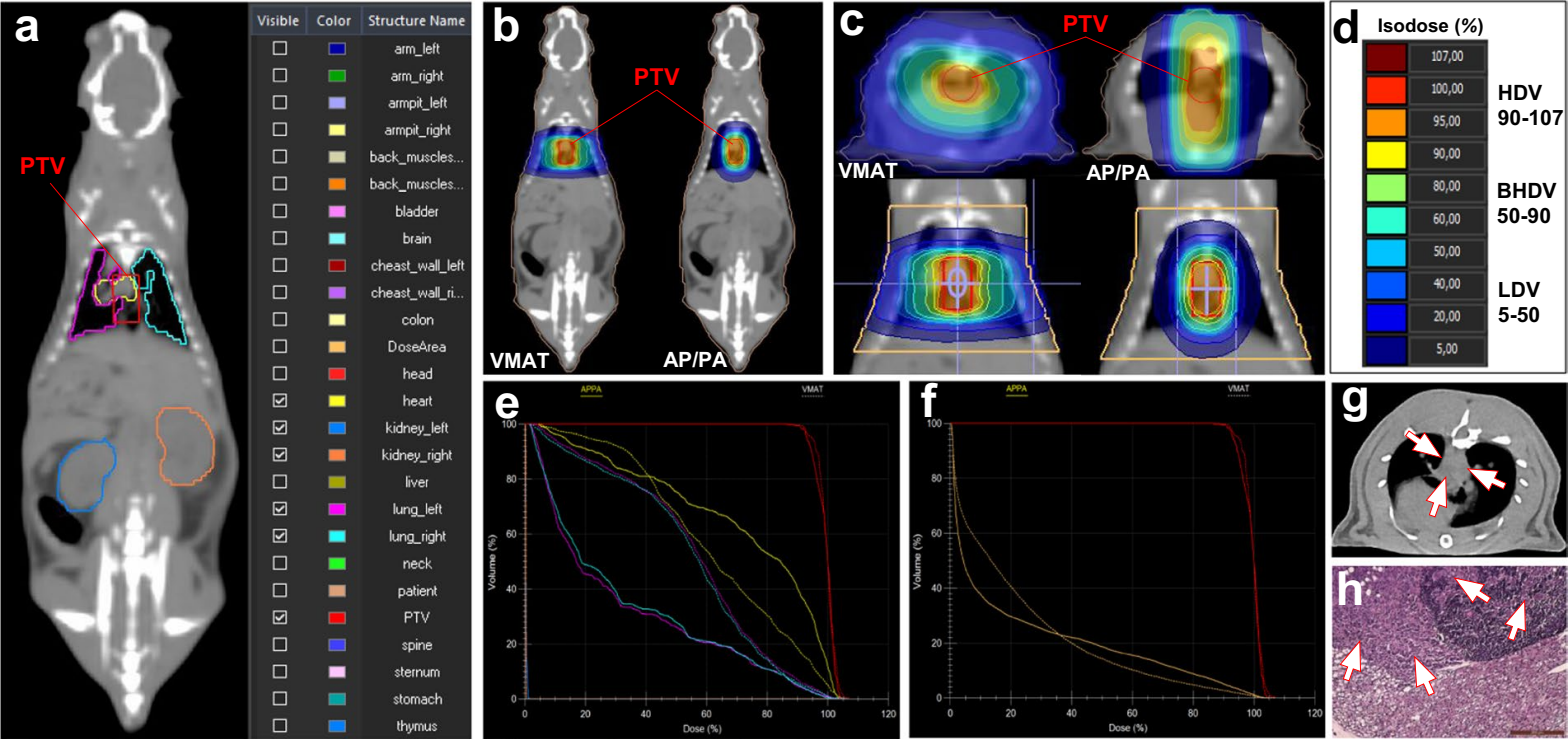
Treatment plans: (**a**) PTV and contoured structures at risk of the rat with selected organs shown (heart, left and right lungs, left and right kidneys). (**b**–**d**) Dose distributions with isodose curves for VMAT and AP/PA irradiation; (**b**,**c**) posterior and transversal views. (**d**) Dose levels for the different dose regions HDV: 90–107%, BHDV: 50–90%, LDV: 5–50%, and NIRV: <5%. (**e**) DVHs for VMAT (solid lines) and AP/PA (dashed lines): PTV (red), heart (yellow), lungs (turquoise/magenta), kidneys (blue/orange: barely visible due to very low integral dose); (**f**) DVHs for the PTV (red) and the thoracic subvolume outside the PTV (brown; ‘patient’); (**g**) tumor detection using a dual-source high-resolution CT scanner (arrows indicate tumor affected tissues); (**h**) histological section of tumor (malignant mesothelioma) invading the bordering lymphatic gland and connective tissues (H&E stain; scale bar: 200 µm). Abbreviations: DVHs, dose-volume histograms; PTV, planning target volume; VMAT, volumetric-modulated arc therapy; AP/PA, anterior-posterior/posterior-anterior; Gy, Gray; AN/CBCT, anesthesia only and cone-beam computed tomography only control groups combined, HDV, high-dose volume; BHDV, bordering high-dose volume; LDV, low-dose volume; NIRV, non-irradiated volume; H&E, hematoxilin & eosin.

Radiation treatment planning was based on computed tomography (CT) scans using two representative body sizes, 250 g (large) and 170 g (small) and applied to individual animals according to their weight (Supplementary Table S3). Treatment plans (Monaco^®^, Version 5.0, Elekta, Stockholm, Sweden) for a 296 mm^3^ cylindrical planning target volume (PTV) in the mediastinum with an adapted VMAT 360° arc or two opposite AP/PA beams. The PTV and representative major organs at risk was based on detailed contouring are shown (Fig. 1a). Dose distributions in the whole animal and a subvolume containing the irradiated thorax (Fig. 1b–d) were used to define dose regions and to generate dose-volume histograms (DVHs). Four dose-regions (Fig. 1d) were defined: high-dose volume (HDV, 90–107% of the target doses), bordering high-dose volume (BHDV, 50–90%), low-dose volume (LDV, 5–50%) and non-irradiated volume (NIRV, <5%). The sizes of the different dose volumes relative to the total body volume are shown in Supplementary Fig. S3. The absolute volumes are included in Supplementary Table S4a. The DVHs for the two irradiation techniques showed clear protection of the lungs and to some extent of the heart by VMAT, as well as a very low dose to the kidneys for both techniques (Fig. 1e). To compare the dose distributions in the normal tissue outside the PTV, a smaller volume was defined for the irradiated thorax. This clearly demonstrates the larger low-dose volume and the smaller high-dose volume for VMAT compared with AP/PA (Fig. 1f). Irradiation was performed with 6 megavolt (MV) X-rays from a clinical linear accelerator (LINAC; Versa HD, Elekta AB, Stockholm, Sweden) equipped with a multi-leaf collimator with 160 interdigitating leaves with 0.5 cm beam width at the isocenter. Three dose fractions (3 × 5 Gy or 3 × 8 Gy) were delivered every other day after CBCT positioning over a period of five days to minimize negative physiological consequences of anesthetizing the animals on consecutive days. The equivalent total doses given in daily fraction sizes of 2 Gy/fraction (EQD2) were equal to 19 Gy and 36 Gy respectively (calculated from the linear-quadratic model with α/β = 10 Gy), i.e. comparable with the 20–30 Gy recommended by the German Hodgkin Study Group (GHSG).

### Tumor detection and pathology

Rats were monitored daily for up to 431 days and underwent a weekly evaluation for early signs of sickness (body weight loss >10%, color change, aberrant behavior, etc.). Animals with suspected tumors were examined with a dual-source, high-resolution (spatial resolution 0.24 mm) and ultra-low dose (<50% compared to conventional systems) CT scanner (SOMATOM Force, Siemens Healthcare Diagnostics GmbH, Eschborn, Germany) delivering high-quality chest images, even at the fast respiratory and coronary activity (60–170 breaths/min, 300–500 beats/min) of rats. Representative CT images are shown in Fig. 1g and Supplementary Fig. S4. If a tumor was detected, animals were sacrificed by CO_2_ inhalation for subsequent standardized autopsy. Entire organs and tissues (including lungs, heart, thymus, trachea, esophagus, large thoracic blood vessels, diaphragm, chest wall with ribs, thoracic vertebrae as well as liver, spleen, pancreas, intestine, and bladder), and tumors, were fixed in 3.7% formalin (pH 7.4) for ≥48 h. 3–4 µm thick histological sections (Fig. 1h) were prepared for hematoxylin and eosin (H&E) staining (LEICA Autostainer XL, Leica Biosystems, Nussloch, Germany) and microscopy (LEICA DMBRE upright light microscope, Leica Microsystems, Wetzlar, Germany). Small pieces of tumors from a subset of *n* = 40 rats representative for all treatment groups and dose regions were shock-frozen and preserved at −80 °C for histology and determination of LOH (*Tp53*). Inflammation was scored in irradiated mediastinal tissues of *n* = 19 rats with tumors in the NIRV of irradiated groups and AN/CBCT controls (*n* = 22) based on focal lymphocyte agglomerations: 0 (none), 1 (rare), 2 (moderate), and 3 (frequent).

### Statistical analysis

Differences in proportions were compared using Fisher’s exact test. The latency time (time-to-tumor, TTT, appearance leading to sacrifice of the animal) was visualized by Kaplan-Meier survival curves and differences were tested for significance using the Gehan-Breslow-Wilcoxon test. Hazard ratios (HR) and 95% confidence intervals (c.i.) for proportional hazards were determined by the logrank method. TTT differences between groups were tested using the Mann-Whitney U-test. GraphPad Prism version 7 (GraphPad Software Inc., San Diego, CA, USA) and JMP statistical Discovery Software version 13 (SAS Institute Inc., Böblingen, Germany) were used for the analyses.

## Results

### Tumor induction

**Figure 2:**
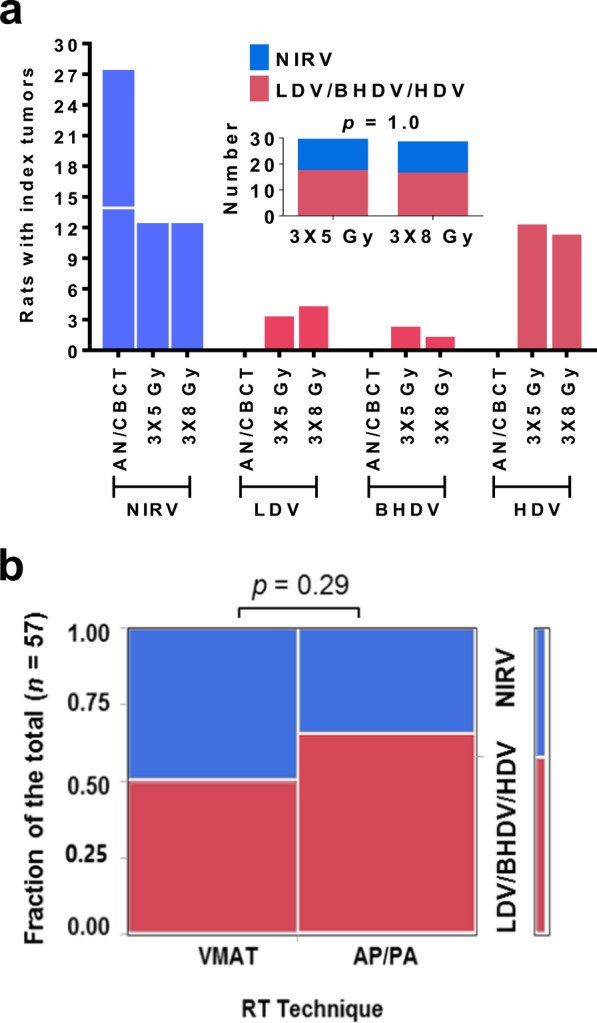
Tumor development in unirradiated and irradiated volumes: (**a**) control animals (AN/CBCT) developed spontaneous tumors only in the body parts equivalent to the NIRV of irradiated animals whereas only irradiated animals developed tumors in the LDV, BHDV and HDV, and the distribution of tumors in irradiated and unirradiated volumes was similar for the two prescribed doses, 3 × 5 Gy and 3 × 8 Gy; (**b**) no significant difference in the proportion of radiation-associated tumors (in the combined LDV/BHDV/HDV) was observed between the two RT techniques, VMAT and AP/PA (*p* = 0.29; Fisher’s exact test; α < 0.05). Abbreviations: NIRV, non-irradiated volume; LDV/BHDV/HDV, combined low-dose volume/bordering high-dose volume, and high-dose volume; AN/CBCT, anesthesia only and cone-beam computed tomography only control groups combined; Gy, Gray; RT, radiotherapy; VMAT, volumetric-modulated arc therapy; AP/PA, anterior-posterior/posterior-anterior irradiation.

Spontaneous or radiation-associated tumors could be analyzed in *n* = 84 animals (13–15 rats per group) while six rats were sacrificed due to other causes (Table 1). In the two control groups (AN, CBCT), all tumors (*n* = 27) were found in the body volume corresponding to the NIRV in the irradiated animals. By contrast, more than half of the tumors in irradiated animals were found in the HDV, BHDV or LDV (17/29 and 16/28 after 3 × 5 and 3 × 8 Gy, respectively). The number of tumors in the NIRV and irradiated volumes were similar in the 3 × 5 Gy and 3 × 8 Gy groups (Fig. 2a). No evidence for an increased induction of tumors in the irradiated volumes after VMAT compared to AP/PA was found (14/28 vs. 19/29; *p* = 0.29, Fisher’s exact test; Fig. 2b). Of the 33 tumors in the irradiated volumes, the majority (*n* = 26) developed in volumes receiving >50% of the target dose (HDV: *n* = 23, BHDV: *n* = 3) and only a minority (*n* = 7) in the LDV. The yield of radiation-associated tumors per cm^3^ estimated from the treatment plans was approximately 30-fold higher in the HDV compared with the LDV and BHDV (Supplementary Table S4).

**Table 1:** Number of rats with tumors in the NIRV, and different RT planning volumes (LDV, BHDV, HDV), for each group (dose level and treatment modality).

Treatment group	NIRV	LDV	BHDV	HDV	Total
AN	13	—	—	—	13
CBCT	14	—	—	—	14
VMAT 3 × 5 Gy	5	2	1	7	15
AP/PA 3 × 5 Gy	7	1	1	5	14
VMAT 3 × 8 Gy	9	—	—	4	13
AP/PA 3 × 8 Gy	3	4	1	7	15
Totals	51	7	3	23	84

Abbreviations: NIRV, non-irradiated volume; LDV, low-dose volume; BHDV, bordering high-dose volume; HDV, high-dose volume; AN, anesthesia only controls; CBCT, cone-beam computed tomography only controls; VMAT, volumetric-modulated arc therapy; AP/PA, anterior-posterior/posterior-anterior irradiation; Gy, Gray.

### Tumor latency and dose levels

**Figure 3:**
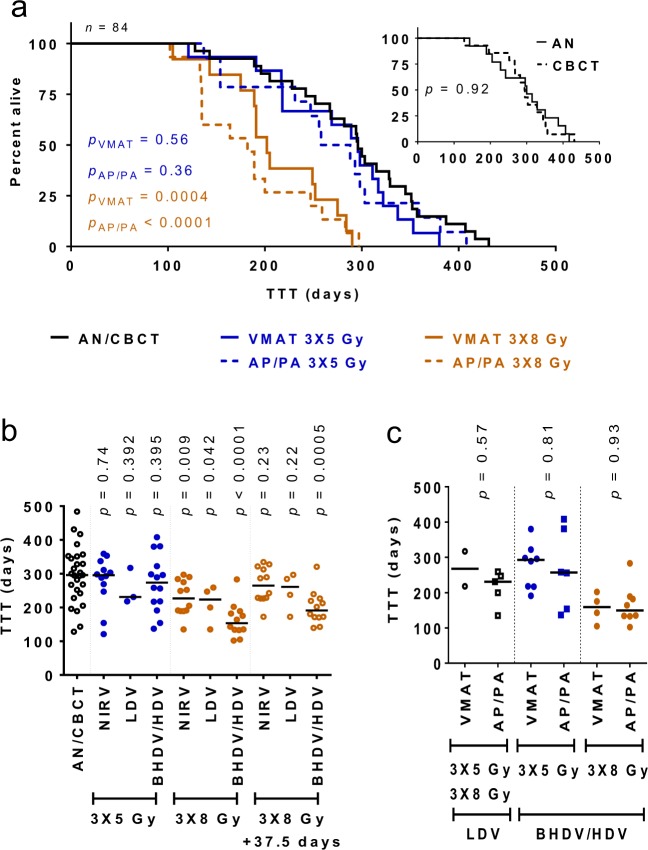
Latency time of tumor development: (**a**) Kaplan-Meier curves showing the fraction of tumor-free animals as function of time after irradiation (or sham) for all animals that eventually developed a tumor (*n* = 84). The curves for the two control groups (AN and CBCT) were similar (*p* = 0.92; **a**, insert), but tumors developed significantly earlier after 3 × 8 Gy (VMAT: *p* = 0.0004, and AP/PA: *p* < 0.0001; Gehan-Breslow-Wilcoxon test; *p* < 0.05); (**b**) the median time to tumor (TTT) remained significantly reduced in the BHDV/HDV (*p* = 0.0005; Mann-Whitney U test) when the older age at treatment of the 3 × 8 Gy group (median 37.5 days) was considered; (**c**) no significant difference in the TTT for radiation-associated tumors (in LDV/BHDV/HDV) was observed between VMAT and AP/PA. Horizontal lines indicate median values. Abbreviations: TTT, time to tumor; AN, anesthesia only control group; CBCT, cone-beam computed tomography only control group; VMAT, volumetric-modulated arc therapy; AP/PA, anterior-posterior/posterior-anterior irradiation; Gy, Gray; NIRV, non-irradiated voume; LDV, low-dose volume; BHDV/HDV, bordering high-dose volume and high-dose volume combined.

Kaplan-Meier curves for the AN and CBCT control groups were largely identical (median 300 vs. 294.5 days; *p* = 0.92) indicating that the effect of the low-dose exposure during the positioning CBCT scans on tumor induction (HR = 1.053, 95% c.i. = 0.497–2.25) was negligible (Fig. 3a). Treatment with 3 × 8 Gy led to a significantly earlier tumor appearance for both modalities (VMAT: median 202 days; *p* = 0.0004; AP/PA: median 182 days; *p* < 0.0001) vs. the combined AN/CBCT control groups (median 296 days). This was corroborated by proportional hazards analysis (HR = 3.24, c.i. = 3.01–19.1 for VMAT and HR = 3.56, c.i. 3.75–22.0 for AP/PA). Detailed analysis of the TTT showed a statistically significant decrease for tumors within the HDV/BHDV after 3 × 8 Gy compared to AN/CBCT (median 169.5 vs. 296 days; *p* < 0.0001) but not 3 × 5 Gy (Fig. 3b). After adding 37.5 days to compensate for the higher median age at treatment of the 3 × 8 Gy groups, the lower TTT in the HDV/BHDV remained significant (median 191 days vs 296 days for AN/CBCT; *p* = 0.0005) whereas it was not significant for tumors in the LDV and NIRV. Importantly, no significant difference in TTT was detected between VMAT and AP/PA treatments for any dose level (LDV: median 267.5 vs. 231 days, *p* = 0.57; 3 × 5 Gy, HDV/BHDV: median 292.5 vs. 257 days; *p* = 0.81; 3 × 8 Gy, HDV/BHDV: median 159 vs. 149.5 days; *p* = 0.93; Fig. 3c).

### Tumor spectra

**Figure 4:**
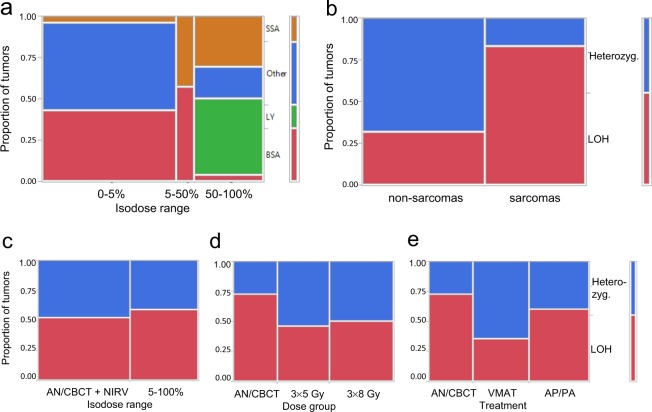
Spectrum of index tumors in different dose ranges (<5% includes unirradiated controls and irradiated groups): (**a**) SSA was strongly associated with irradiation (*p* = 0.0001), and LY developed only in the HDV (90–107%). The index tumors from all *n* = 84 animals were included; (**b**) LOH was very frequent in sarcomas but only occurred in a minority of non-sarcoma tumors (*p* = 0.0015); (**c**–**e**) LOH did not depend on the dose region (*p* = 0.75), prescribed target dose (*p* = 1.00) or RT treatment technique (*p* = 0.27), Fisher’s exact tests (α < 0.05). *Abbreviations*: SSA, soft sarcoma, LY, lymphoma; BSA, bone sarcoma; LOH, loss of heterozygosity (+/C273X); AN/CBCT/NIRV, combined anesthesia only and cone-beam computed tomography only control groups and non-irradiated volume; LDV, low dose volume; Gy, Gray; VMAT, volumetric-modulated arc therapy; AP/PA, anterior-posterior/posterior-anterior irradiation.

Pathological examination of tumors showed that the distribution of tumor entities in the NIRV of irradiated groups resembled that found in the unirradiated control groups with the major entities being bone sarcoma and non-sarcoma tumors (Table 2). Combining the NIRV of irradiated animals and the control groups, the most frequent entities were bone sarcoma (*n* = 22) and carcinoma (*n* = 8), while soft tissue sarcoma (*n* = 2), and brain tumors (*n* = 3), were also detected but no lymphoma. No breast cancers were found in the thoracic glands whereas breast cancer were detected in the inguinal glands (corresponding to the NIRV, i.e. outside the irradiated field) in two control group animals (*n* = 14 females) and one animal in the 3 × 8 Gy VMAT group (n = 29 females in all irradiated groups). Atypical morphology prevented exact pathological identification in *n* = 13 rats with pelvic and abdominal (non-osseous) tumors. In the irradiated volumes, the most frequently detected tumor entity was lymphoma (*n* = 12), followed by soft tissue sarcoma (*n* = 11), bone sarcoma (*n* = 5), carcinoma (*n* = 3), and malignant mesothelioma (*n* = 2). The tumor type distribution was significantly different between dose ranges (*p* < 0.0001; Fig. 4a). Thus soft tissue sarcoma and lymphoma were strongly associated with the irradiated volumes (*n* = 11 soft tissue sarcomas were detected in the HDV/BHDV/LDV vs. *n* = 2 at isodoses <5%, *p* = 0.0001, while *n* = 12 lymphomas were detected only in the HDV).

**Table 2:** Number and entity of index tumors per volume. Bone sarcoma and carcinoma were frequent in unirradiated controls and non-irradiated volumes (NIRVs) of irradiated rats while lymphoma and soft tissue sarcoma were frequent in combined irradiated low-dose volume, bordering high-dose volume and high-dose volume (LDV/BHDV/HDV).

Volume	BSA	CA	SSA	BT	BC	LY	MM	ND	Total	
									♀	♂
AN/CBCT	13	4	1	2	2	—	—	5	14	13
NIRV 3 × 5 Gy/3 × 8 Gy combined	9	4	1	1	1	—	—	8	11	13
VMAT 3 × 5 Gy LDV/BHDV/HDV	3	1	2	—	—	3	1	—	5	5
AP/PA 3 × 5 Gy LDV/BHDV/HDV	—	1	2	—	—	4	—	—	4	3
VMAT 3 × 8 Gy LDV/BHDV/HDV	—	—	1	—	—	2	1	—	2	2
AP/PA 3 × 8 Gy LDV/BHDV/HDV	2	1	6	—	—	3	—	—	7	5
Total	27	11	13	3	3	12	2	13	43	41

Abbreviations: BSA, bone sarcoma; CA, carcinoma, SSA, soft sarcoma; BT, brain tumor; BC, breast cancer; LY, lymphoma; MM, malignant mesothelioma; ND, not determined; NIRV, non-irradiated volume; Gy, Gray; AN, anesthesia only controls; CBCT, cone-beam computed tomography controls; VMAT, volumetric-modulated arc therapy; AP/PA, anterior-posterior/posterior-anterior irradiation; LDV/BHDV/HDV, low-dose volume, bordering high-dose volume, and high-dose volume combined (planned volume).

In six animals, microscopic evaluation revealed an additional tumor within sections containing the index tumor in the LDV/BHDV/HDV (Supplementary Table S5). Three of these tumors were located in the high-dose regions HDV/BHDV and three in the LDV. Bone sarcoma was the most common additional tumor found (4/6) and, notably, all were of a different type than the index tumor. Additional carcinoma (*n* = 3) and mesothelioma (*n* = 2) were also found in the NIRV of the rats that already had bone sarcoma (*n* = 3) or carcinoma (*n* = 2) respectively. Radiation-induced lymphoma developed predominantly in males while soft-tissue sarcoma were more frequent in female rats (*p* = 0.0048, Fishers’ exact test; *n* = 28 males and *n* = 29 females in the irradiated groups (Supplementary Fig. S5). No significant differences in TTT were observed between tumor types at the different dose levels (Supplementary Fig. S6).

### LOH of *Tp53*

Sequence analysis of 40 representative index tumors that could be examined for LOH of *Tp53*, revealed LOH in 55% of the tumors, which was strongly associated with sarcomas (bone and soft tissue) in contrast to non-sarcoma tumors (15/18 vs 7/24; *p* = 0.0015, Fisher’s exact test; Fig. 4b). By contrast, no significant differences were found between irradiated and unirradiated volumes (*p* = 0.75), prescribed doses (*p* = 1.00), and treatment modalities (*p* = 0.27; Fig. 4c–e). All LOH showed the C273X mutation without sequence changes, consistent with complete loss of the wildtype allele or duplication of the mutated allele. A weak trend for shorter median TTT (by 45 days; *p* = 0.12) and attained age (by 36 days; *p* = 0.12) was observed for animals with tumors exhibiting LOH (Supplementary Fig. S7).

Pathological examination of mediastinal H&E-stained sections indicated pulmonary inflammatory alterations in form of microscopic inflammatory foci. Most but not all of the lung volume was within the LDV of the irradiated animals but no evidence for increased inflammation (score 2–3) in irradiated vs unirradiated lungs (*p* = 0.20) was observed (Supplementary Table S6 and Supplementary Fig. S8).

## Discussion

This work is the first experimental study testing the dogma that the low-dose bath (‘a little to a lot’) of modern IMRT induces more cancers than conventional treatment, where high(er) doses are applied to smaller volumes of normal tissue (‘a lot to a little’). No significant difference in tumor incidence between VMAT and AP/PA was detected. In both treatment groups, the majority of radiation-associated tumors were found in the mediastinal volume receiving 90–107% of the target dose.

The hypothesis of increased induction of second cancers in the low-dose volume is based on models of radiation-induced carcinogenesis assuming a linear dose response that reaches a maximum, or saturation, at moderate doses owing to the combined effect of mutation, cell killing, and repopulation^15–19^. The present results show a much lower rate of tumors in the LDV (receiving a total dose of 0.75–12 Gy) and BHDV (7.5–21.6 Gy) than in the HDV (total doses of 13.5–24 Gy) indicating an increase in the dose-response relationship up to at least 15 Gy. This is consistent with recent analyses of clinical data for localized RT showing a linear dose response for the relative risk of second cancers of the breast, brain, lung, and bone, with the exception of thyroid cancer which showed a maximum at 16–20 Gy^4^. Furthermore, sarcoma after RT for childhood cancer increased with dose up to 40 Gy^20^, and in another study up to at least 50 Gy (given in 2 Gy fractions) for sarcoma, basal cell carcinoma of the skin, meningioma, salivary gland cancer, glioma, and breast cancer^21^. However, to firmly establish the shape of the dose-response curve in the present system further experiments with more animals would be needed.

Laboratory rats are in the young adult life phase from approximately 60 to 120 postnatal days^22^. Therefore, all the rats in all six groups were young adults at the time of treatment and, thus, had a similar background risk for tumor development. A reduced latency time for radiation-associated tumors was statistically significant only after 3 × 8 Gy but the fact that tumors also appeared in the irradiated volume after 3 × 5 Gy before spontaneous tumors developed in the NIRV suggests a minor latency shortening. These results extend earlier findings of shortened tumor latency after whole-body irradiation of mice with 1–4 Gy which may be related to induction of an inflammatory microenvironment^23–25^. However, in the present system, the rate of inflammatory foci in the irradiated lungs (largely in the LDV) was not increased and even showed a trend for a decrease after irradiation.

Lymphoma and soft tissue sarcoma were strongly associated with the irradiated volume but mesotheliomas and carcinomas were also found. Although bone sarcoma was the most frequent tumor entity in the NIRV and sham NIRV (AN/CBCT), non-sarcoma tumor entities and tumors of unidentified histology constituted more than half the tumors in the NIRV/AN/CBCT. This contrasts with previous findings from this *Tp53* knockout model, in which bone sarcoma was the dominant tumor type in heterozygote rats^12,26^. The difference in the spectra of tumor entities may be related to minor variations in genetic background which is known to have a strong influence on the type of tumors formed^14,27^. The appearance of lymphoma solely in the HDV might be explained by co-irradiation of the thymus. Radiation-induced lymphoma predominantly developed in male rats while soft tissue sarcoma was more frequent in female rats. Since male rats were larger than females, more thoracic tissue, including the thymus, was exposed to high radiation doses in males compared with females. The increase of soft tissue sarcoma is consistent with the association of such SC with the target volume after radiotherapy^20^. These findings support the validity of the *Tp53* knockout rat model for detecting a range of different radiation-induced tumor entities. Although four of the six thoracic (but none of the six inguinal) mammary glands in the rat were inside the LDV in VMAT planning, no breast cancers arose after irradiation. By contrast, one spontaneous inguinal breast cancer was found in the NIRV of the irradiated groups and two in the corresponding volume in the control groups. Notably, the latter were detected in the AN and not in the CBCT group receiving a very low dose.

The *Tp53*^C273X^ rat model used here showed no detectable truncated p53 which makes it similar to other *Tp53* knockout models in mice^14^ and rats^27–30^. The different spectra of spontaneous tumors in these models are most likely determined by the different genetic background^14,27^. Li-Fraumeni syndrome in humans differ, as cells usually produce full-length *TP53* transcripts, and are frequently heterozygous for a functionally inactivating mutation in the DNA binding domain^31^. By contrast, heterozygous *Tp53*^+/C273X^ rats are expected to show haplo-insufficiency of the p53, which may compromise its role in maintaining genomic stability without producing a gain-of-function frequently associated with mutated *TP53* in human tumors^14,32^. In the present study, *Tp53*^+/C273X^ LOH was mainly associated with sarcomas but not with irradiation. This is consistent with the high rate of *TP53* mutations and LOH in human radiation-induced sarcomas suggesting that p53 inactivation is an early event in many of these tumors^20,33^. Tumor-prone experimental models have the advantage of increasing the rate of tumor induction thus requiring smaller numbers of animals but are limited by the shorter observation time and possible bias of different tumor spectra and genetic background compared with radiotherapy patients. However, these limitations are not expected to seriously influence the difference between VMAT and AP/PA.

In summary, the present results do not support the hypothesis that larger low-dose volumes from IMRT increase the risk of second cancer and instead suggest a strongly increased risk of SC per unit volume at higher doses. This corroborates recent evidence from localized radiotherapy^4,9–11^ and does not support classical radiation carcinogenesis models assuming a linear dose-response relationship up to 4–8 Gy with no further increase at higher doses^8,15^. In the present system, the appearance of spontaneous tumors limits the observation time and reduces the number of radiation-induced tumors that may be observed. To overcome this limitation, further experiments with large numbers of wild-type animals will be needed.

Because a reduction of doses and irradiated volumes in the treatment of childhood cancers has led to fewer SC and serious complications within the healthy tissue after radiotherapy^6,34,35^, the reduced high-dose volume, time and monitor units in modern conformal IMRT might decrease the risk of SC and possibly balance the hypothetical increased risk associated with larger low-dose volumes. The present study - together with clinical studies showing that second cancers are predominantly found close to the PTV^9,10^ - suggests that the current practice of withholding dose-sparing radiotherapy with modern fast IMRT techniques in young Hodgkin’s Lymphoma patients may need to be re-evaluated and, therefore, should be re-examined experimentally and clinically.

## Conclusions

The present study shows for the first time that radiation-induced tumors appeared predominantly in regions receiving high doses with no impact of the “low dose bath” created with VMAT. This does not support the hypothesis that IMRT may increase the risk of second cancer compared to 3D-CRT. Although further experimental studies are called for, our results provide a strong biological rationale for reconsidering the clinical practice of withholding high-dose-sparing IMRT from children and young adults with Hodgkin^,^s Lymphoma.

## Supplementary information

Radiation-induced malignancies after intensity-modulated versus conventional mediastinal radiotherapy in a small animal model

## Data Availability

The datasets from this study are available from the corresponding author on reasonable request.

## References

[1] Curtis, R. E. *et al*. (eds). *New Malignancies Among Cancer Survivors: SEER Cancer Registries, 1973–2000*. (National Cancer Institute. NIH Publ. No. 05-5302. Bethesda, MD, 2006).

[2] Oeffinger KC, Baxi SS, Friedman DN, Moskowitz CS (2013). Solid Tumor Second Primary Neoplasms: Who is at Risk, What Can We Do?. Semin Oncol.

[3] Berrington de Gonzalez A (2011). Proportion of second cancers attributable to radiotherapy treatment in adults: a cohort study in the US SEER cancer registries. Lancet Oncol.

[4] Berrington de Gonzalez A (2013). Second solid cancers after radiation therapy: a systematic review of the epidemiologic studies of the radiation dose-response relationship. Int J Radiat Oncol Biol Phys.

[5] Omer B (2012). Patterns of subsequent malignancies after Hodgkin lymphoma in children and adults. Br J Haematol.

[6] Turcotte LM (2017). Temporal Trends in Treatment and Subsequent Neoplasm Risk Among 5-Year Survivors of Childhood Cancer, 1970–2015. Jama.

[7] Hall EJ (2006). Intensity-modulated radiation therapy, protons, and the risk of second cancers. Int J Radiat Oncol Biol Phys.

[8] Hall EJ, Wuu CS (2003). Radiation-induced second cancers: the impact of 3D-CRT and IMRT. Int J Radiat Oncol Biol Phys.

[9] Diallo I (2009). Frequency distribution of second solid cancer locations in relation to the irradiated volume among 115 patients treated for childhood cancer. Int J Radiat Oncol Biol Phys.

[10] Dorr W, Herrmann T (2002). Second primary tumors after radiotherapy for malignancies. Treatment-related parameters. Strahlenther Onkol.

[11] Tukenova M (2011). Radiation therapy and late mortality from second sarcoma, carcinoma, and hematological malignancies after a solid cancer in childhood. Int J Radiat Oncol Biol Phys.

[12] van Boxtel R (2011). Homozygous and heterozygous p53 knockout rats develop metastasizing sarcomas with high frequency. Am J Pathol.

[13] Smits BM (2006). Generation of gene knockouts and mutant models in the laboratory rat by ENU-driven target-selected mutagenesis. Pharmacogenet Genomics.

[14] Malkin D (2011). Li-fraumeni syndrome. Genes Cancer.

[15] Dasu A, Toma-Dasu I, Olofsson J, Karlsson M (2005). The use of risk estimation models for the induction of secondary cancers following radiotherapy. Acta Oncol.

[16] Sachs RK, Brenner DJ (2005). Solid tumor risks after high doses of ionizing radiation. Proc Natl Acad Sci USA.

[17] Schneider U (2009). Mechanistic model of radiation-induced cancer after fractionated radiotherapy using the linear-quadratic formula. Med Phys.

[18] Shuryak I, Hahnfeldt P, Hlatky L, Sachs RK, Brenner DJ (2009). A new view of radiation-induced cancer: integrating short- and long-term processes. Part II: second cancer risk estimation. Radiat Environ Biophys.

[19] Wheldon EG, Lindsay KA, Wheldon TE (2000). The dose-response relationship for cancer incidence in a two-stage radiation carcinogenesis model incorporating cellular repopulation. Int J Radiat Biol.

[20] Berrington de Gonzalez A, Kutsenko A, Rajaraman P (2012). Sarcoma risk after radiation exposure. Clin Sarcoma Res.

[21] Inskip PD (2016). Radiation-related New Primary Solid Cancers in the Childhood Cancer Survivor Study: Comparative Radiation Dose-response and Modification of Treatment Effects. Int J Radiat Oncol Biol Phys.

[22] Sengupta P (2013). The Laboratory Rat: Relating Its Age With Human’s. Int J Prev Med.

[23] Barcellos-Hoff MH, Nguyen DH (2009). Radiation carcinogenesis in context: how do irradiated tissues become tumors?. Health Phys.

[24] Carlisle SM, Burchart PA, Mitchel RE (2010). Cancer and non-cancer risks in normal and cancer-prone Trp53 heterozygous mice exposed to high-dose radiation. Radiat Res.

[25] Nguyen DH (2011). Radiation acts on the microenvironment to affect breast carcinogenesis by distinct mechanisms that decrease cancer latency and affect tumor type. Cancer Cell.

[26] Hermsen Roel, Toonen Pim, Kuijk Ewart, Youssef Sameh A., Kuiper Raoul, van Heesch Sebastiaan, de Bruin Alain, Cuppen Edwin, Simonis Marieke (2015). Lack of Major Genome Instability in Tumors of p53 Null Rats. PLOS ONE.

[27] Hansen SA (2016). Fischer-344 Tp53-knockout rats exhibit a high rate of bone and brain neoplasia with frequent metastasis. Dis Model Mech.

[28] McCoy A, Besch-Williford CL, Franklin CL, Weinstein EJ, Cui X (2013). Creation and preliminary characterization of a Tp53 knockout rat. Dis Model Mech.

[29] Tong C, Li P, Wu NL, Yan Y, Ying QL (2010). Production of p53 gene knockout rats by homologous recombination in embryonic stem cells. Nature.

[30] Yan HX, Wu HP, Ashton C, Tong C, Ying QL (2012). Rats deficient for p53 are susceptible to spontaneous and carcinogen-induced tumorigenesis. Carcinogenesis.

[31] Kamihara J, Rana HQ, Garber JE (2014). Germline TP53 mutations and the changing landscape of Li-Fraumeni syndrome. Hum Mutat.

[32] Oren M, Rotter V (2010). Mutant p53 gain-of-function in cancer. Cold Spring Harb Perspect Biol.

[33] Gonin-Laurent N (2007). RB1 and TP53 pathways in radiation-induced sarcomas. Oncogene.

[34] Koeck J (2012). Radiotherapy for early mediastinal Hodgkin lymphoma according to the German Hodgkin Study Group (GHSG): the roles of intensity-modulated radiotherapy and involved-node radiotherapy. Int J Radiat Oncol Biol Phys.

[35] LeMieux MH, Solanki AA, Mahmood U, Chmura SJ, Koshy M (2015). Risk of second malignancies in patients with early-stage classical Hodgkin’s lymphoma treated in a modern era. Cancer Med.

