# Supplementary material for: Radiation-induced malignancies after intensity-modulated versus conventional mediastinal radiotherapy in a small animal model
Source: Sci Rep. 2019 Oct 29;9:15489. doi: 10.1038/s41598-019-51735-3 (PMC6820874; doi:10.1038/s41598-019-51735-3)
Supplement: Supplementary file 1 — Radiation-induced malignancies after intensity-modulated versus conventional mediastinal radiotherapy in a small animal model [file 41598_2019_51735_MOESM1_ESM.pdf]

SUPPLEMENTARY INFORMATION

Radiation-induced malignancies after intensity-modulated versus conventional mediastinal radiotherapy in a small animal model

Kaga Gomarteli[#], Jens Fleckenstein[#], Stefanie Kirschner, Vladimir Bobu, Marc A. Brockmann, Thomas Henzler, Mathias Meyer, Philipp Riffel, Stefan O. Schönberg, Marlon R. Veldwijk, Bettina Kränzlin, Christian Hoerner, Gerhard Glatting, Frederik Wenz, Carsten Herskind[*], Frank A. Giordano[*]

[#]equal contributions as first authors; [*]equal contributions as senior authors

**Content**	**Sheet**
Supplementary Methods	2
Supplementary Figure S1	3
Supplementary Table S1	4
Supplementary Table S2	4
Supplementary Figure S2	4
Supplementary Table S3	5
Supplementary Figure S3	5
Supplementary Figure S4	6
Supplementary Table S4	6
Supplementary Table S5	7
Supplementary Figure S5	7
Supplementary Figure S6	8
Supplementary Figure S7	8
Supplementary Table S6	9
Supplementary Figure S8	9

Supplementary methods

Tp53 genotyping and analysis of loss-of-heterozygosity (LOH)

Primers for polymerase chain reaction (PCR) amplification and *Tp53* sequencing (**Supplementary Fig. 1a**) were designed based on the Rnor_6.0 data (INSDC Assembly GCA_000001895.4, July 2014) using standard software (ApE; v2.0.49, M. W. Davis, Utah, USA) and synthesized by Metabion GmbH (Planegg/Stainkirchen, Germany).

Primer set:

Tp53-for1	5´- GCTGAGTATCTGGACGACAGG -3´
Tp53-for2	5´- GTACCGTATGAGCCACCTGAG -3´
Tp53-for3	5'- CGGCCCATCCTTACCATCATC -3´
Tp53-rev2*	5´- AGAAACCACAGCCTCAGAGC -3´
Tp53-rev3*	5´- TGCGCTCTGACGATAATGTCATAG -3´
Tp53-rev4*	5'- GAGAGGAGCTTGTGCTGGTG -3´
Gapdh-for	5´- GGTGAAGGTCGGTGTGAACGG -3´
Gapdh-rev	5´- CCACTTCCAGCCACACTTGCC -3´
Chr10-for	5´- CTTCGGTCTCTTCTCTGACT -3´
Chr10-rev	5´- CAACTGACCGGATAGGATTT -3´

* Reverse primers that bind in intron 7 preventing the amplification of intron-less pseudo-*Tp53* genes.

Extraction of the genomic DNAs (gDNAs) from tissues collected during ear-marking of 5-7 weeks old F1 offspring and from frozen tumor samples was made similarly using a KAPA Express Extract Kit (KAPA Biosystems by Merck AG, Darmstadt, Germany). A KAPA2G Fast PCR Kit (KAPA Biosystems by Merck AG, Darmstadt, Germany) was used for PCR amplification of *Tp53* gDNA fragments from the DNA extracts. Each 1 µl PCR-product was mixed with 6 µl 1× Purple Gel Loading Dye (New England Biolabs, Frankfurt am Main, Germany) for the gel electrophoresis at ~80 mV/116 mA for 1½ h using 1% Agarose/1×TAE gel stained with 5-6 µl Gel Red (INTAS Science Imaging Instruments, Göttingen, Germany) per 100 ml gel. Representative 834 base pair (bp) PCR products (primer pair: Tp53-for2 and Tp53-rev3) and gDNA positive controls (240 bp; Gapdh-for/Gapdh-rev) are shown in **Supplementary Fig. 1b**. For *Tp53* LOH analysis in tumors, 2,605 bp (primer: Tp53-for2/Chr10-rev) or 2,752 bp (primer: Chr10-for/Tp53-rev3) nested PCR products were amplified from 4,523 bp (primer: Chr10-for and Chr10-rev) PCR copies of *Tp53* gene (**Supplementary Fig. 1c**).

For Sanger Sequencing, the following single primers were used: Tp53-for1, Tp53-for2, Tp53-for3, Tp53-rev3 or Tp53-rev4. The *Tp53* fragment containing the C273-encoding TGT triplet that is mutated into TGA in one allele was recognized by similar levels of T/A nucleotide peaks on the sequencing chromatogram, while LOH ($Tp53^{C273X/C273X}$) in tumors was identified by an A/T ratio ≥ 2.0 (**Supplementary Fig. 1d-g**).

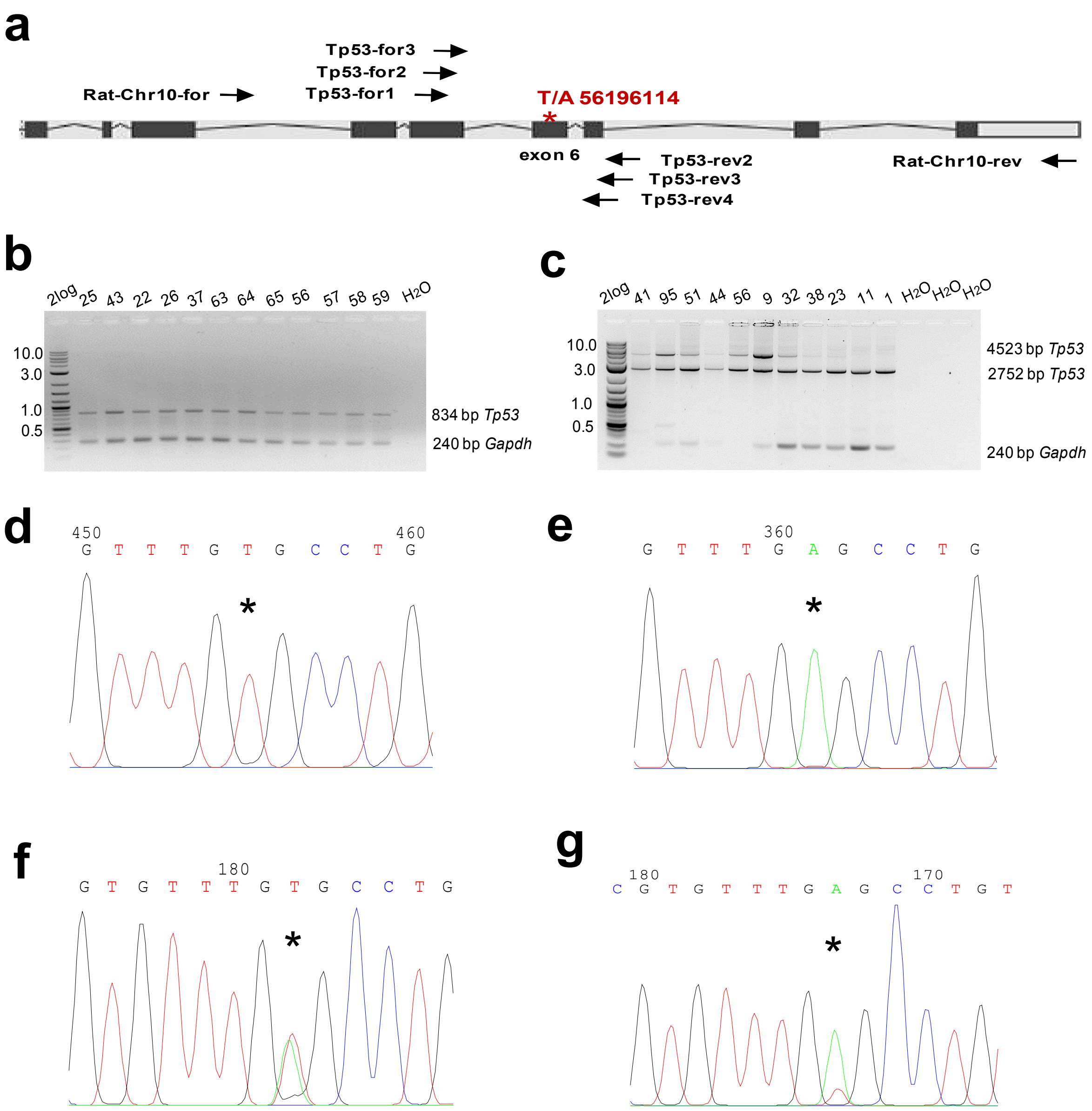

Supplementary Figure S1. Genotyping: (**a**) Schematic representation of the *Tumor protein 53* (*Tp53*) of the rat, containing a T/A 56196114 mutational site in exon 6, with positions of primers for amplifications of genomic DNAs using polymerase chain reaction (PCR); (**b**) representative genotyping PCR copies of the 834 base pair (bp) spanning *Tp53* and 240 bp *Glyceraldehyde-3-phosphate dehydrogenase* (*Gapdh*) fragments together with a DNA-marker (2log) and genomic DNA negative controls (H_2O) (two independent PCR products were mixed before 1.2% agarose gel electrophoresis. The italicized numbers represent the identities of the rats); (**c**) representative amplicons from tumor *Tp53* DNAs (4,523 bp), *Gapdh* (240 bp) and the nested PCR products (2,752 bp), amplified from the 4,523 bp initial PCR products, with appropriated H_2O controls for each PCR (all three separately performed PCR products were mixed prior loading in 1.2% agarose gel. The italicized numbers represent the identities of the rats with appropriated tumors); (**d-g**) exemplary fragments of typical sequencing chromatograms for: (**d**) wild-type ($Tp53^{C273/C273}$: T peak), (**e**) homozygous ($Tp53^{C273X/C273X}$: A peak), (**f**) heterozygous ($Tp53^{+/C273X}$: matched T/A peaks) genotypes or (**g**); LOH expressed as a strongly declined T peak compared to an A peak in tumors (the small T peak may indicate presence of DNA of adjacent normal tissue).

Supplementary Table S1. Distribution of the *Tp53*$^{+/C273X}$ rat litters (L1-L11) among treatment groups in order to balance their ancestral background.

Treatment group	F1			F2								Total
	L1	L2	L3	L4	L5	L6	L7	L8	L9	L10	L11	
AN	4	-	5	2	-	-	-	-	4	-	-	15
CBCT	5	1	4	3	-	-	2	-	-	-	-	15
VMAT 3×5 Gy	6	-	3	1	-	-	4	-	1	-	-	15
AP/PA 3×5 Gy	5	1	3	4	-	-	1	-	1	-	-	15
VMAT 3×8 Gy	-	-	-	-	1	4	-	3	-	4	3	15
AP/PA 3×8 Gy	-	-	-	-	1	5	-	2	-	2	5	15
Total	20	2	15	10	2	9	7	5	6	6	8	90

Supplementary Table S2. Weight (g) and age (days) of female and male animals (*n*=84) at the time of treatment.

Treatment group	Number of rats		Median weight [min-max]		Median age [min-max]
	Females	Males	Females	Males	Females/Males
AN	6	7	162.5 [150-190]	205 [175-305]	82 [63-108]
CBCT	8	6	182.5 [155-200]	272.5 [175-295]	69 [64-96]
VMAT 3×5 Gy	7	8	180 [170-185]	242.5 [225-280]	68 [58-85]
AP/PA 3×5 Gy	9	5	185 [155-225]	250 [225-285]	68 [58-85]
VMAT 3×8 Gy	7	6	215 [185-225]	287.5 [220-335]	106 [81-124]
AP/PA 3×8 Gy	7	8	205 [180-215]	285[215-330]	106 [81-116]

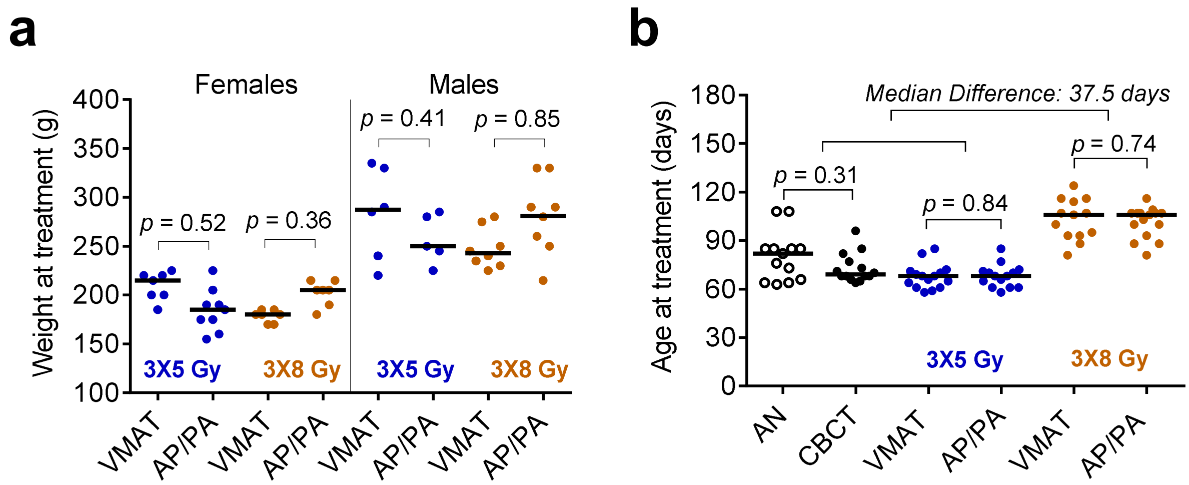

Supplementary Figure S2. Weight and age at treatment among groups: (a) the weight of female and male rats was balanced between VMAT and AP/PA groups at either dose level (3×5 Gy or 3×8 Gy); (b) the median age was 82 days for AN, 69 days for CBCT, 68 days for both 3×5 Gy groups and 106 days for both 3×8 Gy groups; the Mann-Whitney test. The difference between the medians from the combined 3×8 Gy groups and all other groups (pooled) was 37.5 days with insignificant differences between rats recruited to VMAT or AP/PA; plots represent the median; the Mann-Whitney test.

Supplementary Table S3. Recruitment of female and male rats to each radiation treatment group by applied RT-plan size large or small.

Treatment group	Females		Males
	Large (250 g)	Small (170 g)	Large (250 g)
**VMAT 3×5 Gy**	0	7	8
**AP/PA 3×5 Gy**	1	8	5
**VMAT 3×8 Gy**	3	4	6
**AP/PA 3×8 Gy**	0	7	8
**Total**	4	26	27

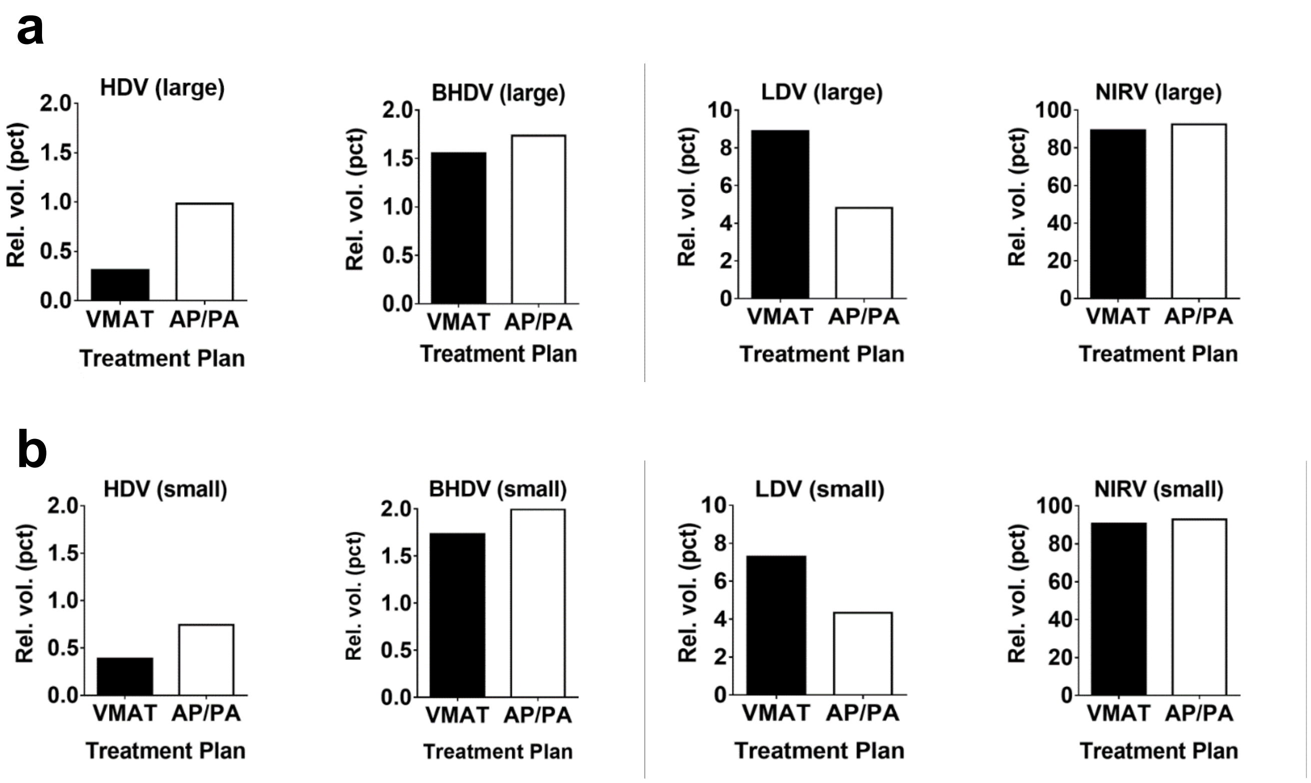

Supplementary Figure S3. Relative volumes of VMAT and AP/PA treatment plans: (a) large and (b) small.

Supplementary Table S4. Tumor yield in volumes at different doses: (**a**) volume in cm^3; (**b**) number of tumors/total number of rats; (**c**) mean number of tumors per rat; (**d**) yield of tumors per cm^3 per rat.

	Dose region	Treatment plan					
		Plan large (250 g)		Plan small (170 g)			
		VMAT	APPA	VMAT	APPA		
**a)**							
	**HDV**	0.782	2.465	0.66	1.265		
	**BHDV**	3.884	4.339	2.946	3.394		
	**LDV**	22.187	12.029	12.394	7.369		
**b)**							
	**HDV**	7/17	6/14	4/11	6/15		
	**BHDV**	1/17	0/14	0/11	2/15		
	**LDV**	0/17	2/14	2/11	3/15		
**c)**							
	**HDV**	0.412	0.429	0.364	0.400		
	**BHDV**	0.059	0.000	0.000	0.133		
	**LDV**	0.000	0.143	0.182	0.200		
**d)**						**Mean yield**	**SD**
	**HDV**	0.527	0.174	0.551	0.316	**3.92E-01**	**1.80E-01**
	**BHDV**	0.015	0.000	0.000	0.039	**1.36E-02**	**1.85E-02**
	**LDV**	0.000	0.012	0.015	0.027	**1.34E-02**	**1.11E-02**

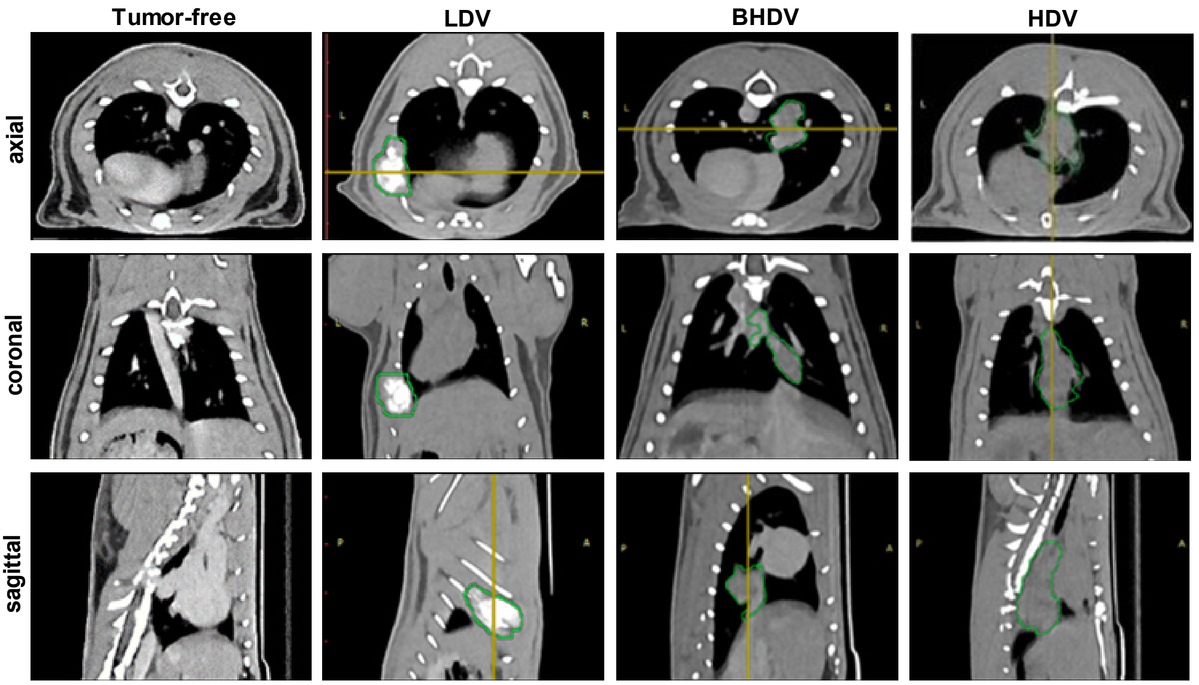

Supplementary Figure S4. Left to right: representative tumor-free thorax of the rat (2 µl/g Imeron-300); native CT images of the solid tumors in the low dose volume (LDV), bordering high dose volume (BHDV), and high dose volume (HDV); green lines define tumor contours.

Supplementary Table S5. Additional tumors in different volumes in irradiated rats and AN-controls.

Additional tumors	Volume	Treatment	Index tumor
Bone sarcoma	HDV-BHDV	VMAT 3×5 Gy	Soft tissue sarcoma
Bone sarcoma	LDV	VMAT 3×5 Gy	Lymphoma
Bone sarcoma	LDV	VMAT 3×5 Gy	Lymphoma
Bone sarcoma	BHDV	AP/PA 3×5 Gy	Lymphoma
Lymphoma	HDV-LDV	AP/PA 3×8 Gy	Soft tissue sarcoma
Carcinoma	BHDV	VMAT 3×8 Gy	Soft tissue sarcoma
Carcinoma	NIRV	VMAT 3×8 Gy	Bone sarcoma
Carcinoma	NIRV	VMAT 3×8 Gy	Bone sarcoma
Carcinoma	NIRV	AN 0 Gy	Bone sarcoma
Mesothelioma	NIRV	AN 0 Gy	Carcinoma
Mesothelioma	NIRV	AN 0 Gy	Carcinoma

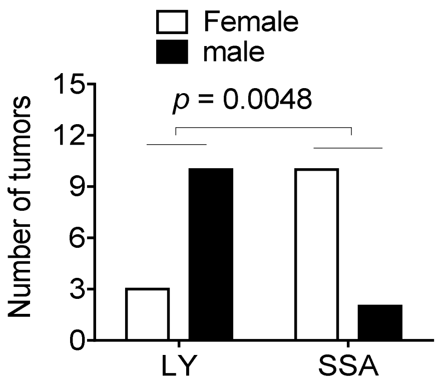

Suppl. Fig. S5. Sex-related differences in the frequency of radiation-associated lymphomas (LY) and sarcomas (SSA): significantly more LY were found in male and more SSA in female rats ($p = 0.0048$, Fisher's exact test). Only irradiated groups were considered (n=28 male and n=29 female rats).

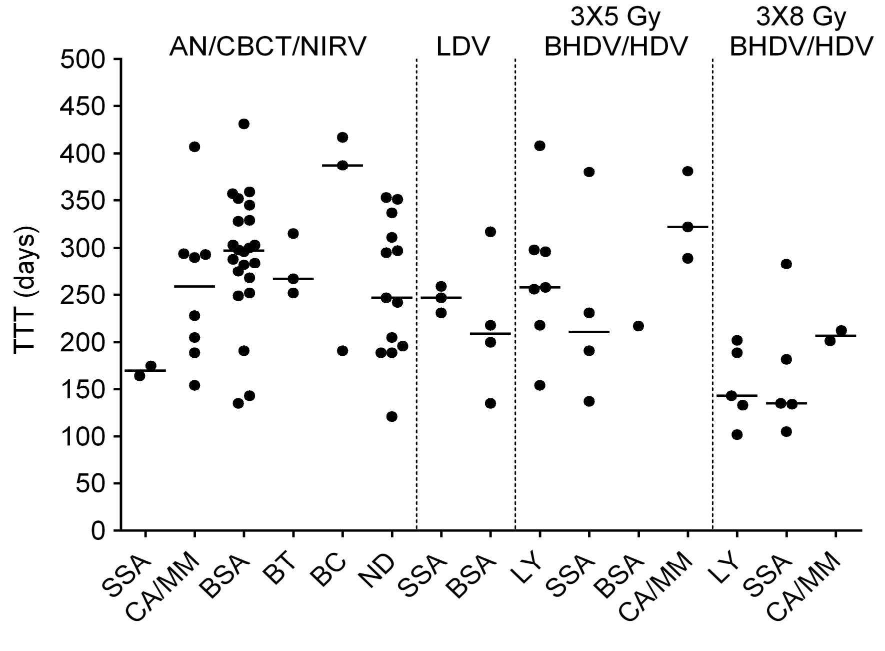

Supplementary Figure S6. Time to tumor (TTT) of different tumor entities: no significant differences in TTT was observed between specific tumor entities. Lymphoma (LY) and soft tissue sarcoma (SSA) were the most common tumors in the bordering high dose volume and high dose volume (BHDV/HDV), whereas most bone sarcomas (BSAs) spontaneously occurred in anesthesia and cone-beam CT controls (AN/CBCT), and in the non-irradiated volume (NIRV) of irradiated rats. Most of BSA and carcinoma (CA), but also malignant mesothelioma (MM), brain tumor (BT), breast cancer (BC), and not determined (ND) tumors, were found in controls and rats developing tumors in the NIRV.

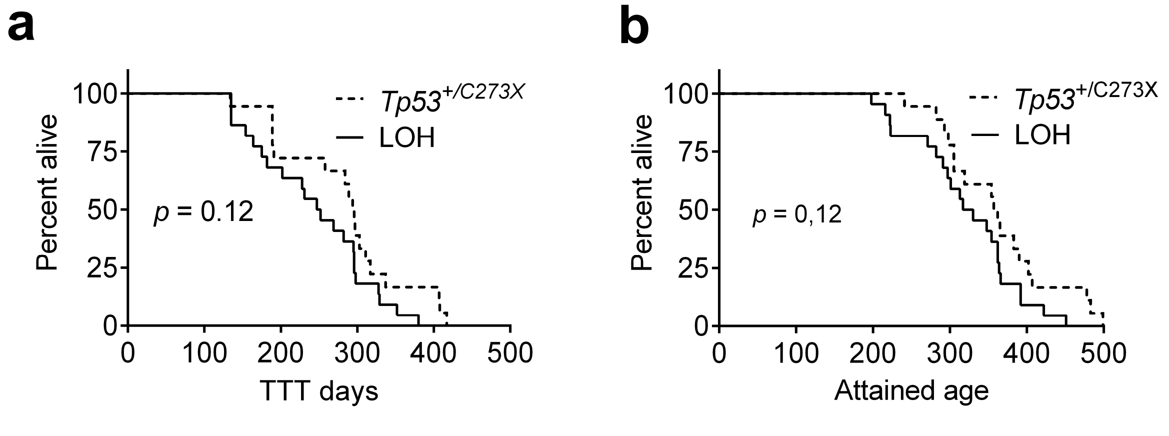

Supplementary Figure S7. Kaplan-Meier curves for tumors with and without Lloss of heterozygosity (LOH) and the time to tumors (TTT): non- significant trends for lower median TTT (a) and attained age (full lifespan; b) were found for tumors with LOH (Gehan-Breslow-Wilcoxon test).

Supplementary Table S6. Inflammatory cells in irradiated and unirradiated rat lungs: none (0), rare (1), moderate (2) and frequent (3) inflammation based on the number and expansion of the inflammatory foci (lymphocyte cluster) in irradiated and unirradiated lungs.

Treatment group	Inflammation grading			
	Grade 0	Grade 1	Grade 2	Grade 3
**AN**	2	4	2	3
**CBCT**	3	2	6	-
**VMAT 3×5 Gy**	1	1	1	-
**AP/PA 3×5 Gy**	3	1	-	-
**VMAT 3×8 Gy**	1	5	3	-
**AP/PA 3×8 Gy**	1	1	1	-

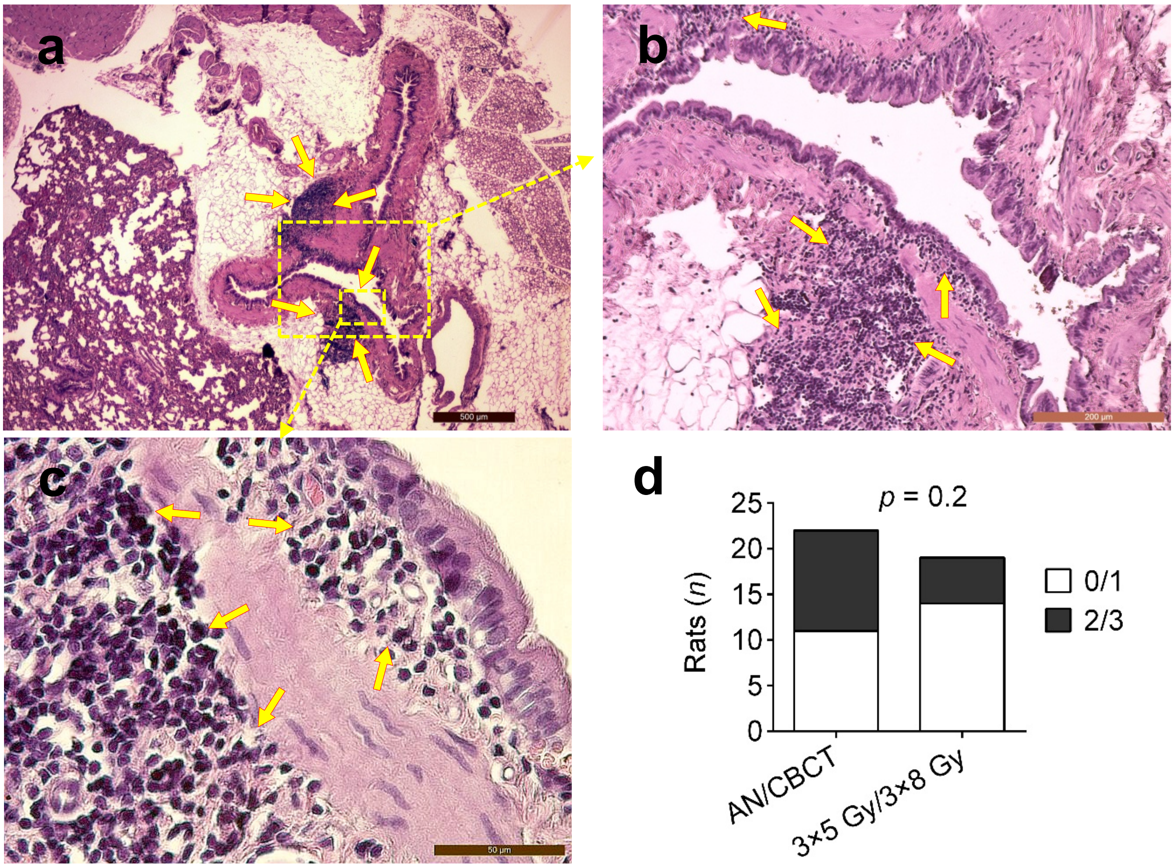

Supplementary Figure S8. Inflammation in a tumor-free mediastinal cross-section determined by microscopy. (**a** - **c**) H&E stained 4 µm thick slide with a segmental bronchus, surrounding mesothelium, and lung tissues; arrows indicate lymphocyte foci; scale-bars: (**a**) 500 µm, (**b**) 200 µm, and (**c**) 50 µm; (**d**) no significant trend was observed for a lower proportion of inflammatory sites grade 2 and 3 in irradiated compared with control animals (Fisher's exact test).
